# Early Diagnosis of Hepatocellular Carcinoma Using Machine Learning Method

**DOI:** 10.3389/fbioe.2020.00254

**Published:** 2020-03-27

**Authors:** Zi-Mei Zhang, Jiu-Xin Tan, Fang Wang, Fu-Ying Dao, Zhao-Yue Zhang, Hao Lin

**Affiliations:** Key Laboratory for Neuro-Information of Ministry of Education, School of Life Sciences and Technology, Center for Informational Biology, University of Electronic Science and Technology of China, Chengdu, China

**Keywords:** hepatocellular carcinoma, early diagnosis, cirrhosis, REOs, mRMR, support vector machine

## Abstract

Hepatocellular carcinoma (HCC) is a serious cancer which ranked the fourth in cancer-related death worldwide. Hence, more accurate diagnostic models are urgently needed to aid the early HCC diagnosis under clinical scenarios and thus improve HCC treatment and survival. Several conventional methods have been used for discriminating HCC from cirrhosis tissues in patients without HCC (CwoHCC). However, the recognition successful rates are still far from satisfactory. In this study, we applied a computational approach that based on machine learning method to a set of microarray data generated from 1091 HCC samples and 242 CwoHCC samples. The within-sample relative expression orderings (REOs) method was used to extract numerical descriptors from gene expression profiles datasets. After removing the unrelated features by using maximum redundancy minimum relevance (mRMR) with incremental feature selection, we achieved “11-gene-pair” which could produce outstanding results. We further investigated the discriminate capability of the “11-gene-pair” for HCC recognition on several independent datasets. The wonderful results were obtained, demonstrating that the selected gene pairs can be signature for HCC. The proposed computational model can discriminate HCC and adjacent non-cancerous tissues from CwoHCC even for minimum biopsy specimens and inaccurately sampled specimens, which can be practical and effective for aiding the early HCC diagnosis at individual level.

## Introduction

Liver cancer is the fourth leading cause of death in patients with malignant cancerous (Indhumathy et al., 2018; Villanueva, 2019). Hepatocellular carcinoma (HCC), which accounts for approximately 90% of all liver cancer cases, is frequently diagnosed at a late stage and has a poor prognosis. Thus, the early HCC diagnosis is significant to improve the prognosis and survival of patients (Asia-Pacific Working Party on Prevention of Hepatocellular Carcinoma, 2010). At present, diagnosis of HCC is based on laboratory investigations and imaging techniques (El-Serag, 2011; Hartke et al., 2017). Nevertheless, for HCC, especially for early HCC, current serum biomarkers and tools, such as α-fetoprotein (AFP) and imaging techniques, displayed poor diagnostic sensitivity and specificity (Sun et al., 2015). Liver biopsy is regarded as a good diagnostic choice in clinical practice only when imaging techniques cannot provide accurate identification of HCC (Russo et al., 2018). However, the biopsy location is usually inaccurate, which might result in inaccurately sampling and thus decrease the diagnosis successful rate (Forner et al., 2008). Therefore, it is necessary to design new methods or discovery new diagnostic signatures to assist the pathologists in the identification of early HCC using biopsy specimens, even inaccurately sampled biopsy specimens. It is likely that the adjacent non-cancerous tissues (cirrhosis tissues in patients with HCC or normal tissues in patients with HCC) can be affected by cancerous tissues, so that they may obtain some similar molecular characteristics of cancerous tissues (Budhu et al., 2006; Wei et al., 2014).

The existed diagnostic signatures are mainly on the basis of risk scores obtained from signature genes’ expression (Wurmbach et al., 2007; Archer et al., 2009; Zhou et al., 2015, 2017; Qu et al., 2019), which are highly sensitive to measurement batch effects (Guan et al., 2018) and are hardly applied in clinical settings. Luckily the relative expression orderings (REO)-based strategy (Zhang et al., 2013; Zhou et al., 2013; Wang et al., 2015; Li et al., 2016), which was firstly proposed by Eddy et al. (2010), is highly robust against experimental batch effects (Cai et al., 2015; Ao et al., 2016; Zhao et al., 2016) and platform differences (Guan et al., 2016), partial RNA degradation (Chen et al., 2017; Liao et al., 2017, 2018; Tang et al., 2018) and uncertain sampling sites within the same cancer tissue (Cheng et al., 2017). And thus the REOs have been used in the early diagnosis of HCC (Ao et al., 2018), gastric cancer (Yan et al., 2019) and colorectal cancer (Guan et al., 2019). In 2018, Ao et al. (2018) obtained 19 gene pairs by using the within-sample REOs. These genes could improve early HCC diagnosis using biopsy specimens, even inaccurately sampled biopsy specimens. However, the rule to identify HCC based on REOs is so simply that some intrinsic relationships among these genes are not revealed. Moreover, the accuracy for HCC diagnosis should still be improved.

Machine learning method is a good choice to uncover underlying patterns (Stephenson et al., 2019). It has been widely employed in bioinformatics (Cao et al., 2017; Bao et al., 2019; Conover et al., 2019; Moritz et al., 2019; Stephenson et al., 2019; Zou and Ma, 2019; Sun et al., 2020). The current work aims to develop a machine learning based method to diagnose HCC within-sample REOs. By removing redundant REOs using minimum redundancy maximum relevance (mRMR), a diagnostic signature consisting of 11 gene pairs was obtained. These signatures were also applied in some independent datasets for examining the performance of these gene pairs for HCC identification. High accuracies were obtained, suggesting that the obtained 11-gene-pair signature based on mRMR is better than the existed 19-gene-pair signature gained by Ao et al. (Ao et al., 2018).

## Materials and Methods

### Data Collection and Preprocessing

The gene expression profiles datasets were freely gained from GEO (Barrett et al., 2005) and TCGA (Tomczak et al., 2015) database. Firstly, according to the type and sampling method of samples, the training datasets were derived from biopsy samples of HCC (D1), surgery samples of HCC (D2), biopsy samples of CwoHCC (D3), and surgery samples of CwoHCC (D4), respectively. To objectively evaluate the model, we separated the samples of each type (D1, D2, D3, and D4) mentioned above into two data subsets: training (80% samples of each type) and testing datasets (20% samples of each type). Finally, the training datasets contained 1091 HCC samples (112 biopsy samples of HCC and 979 surgery samples of HCC) and 242 CwoHCC samples (70 biopsy samples of CwoHCC and 172 surgery samples of CwoHCC). The testing datasets contained 73 biopsy samples (29 HCC samples and 44 CwoHCC samples) and 263 surgery samples (245 HCC samples and 18 CwoHCC samples). The independent datasets, which was comprised of surgical resection samples and biopsy samples, was used to evaluate the performance signature. We used the R package of TCGAbiolinks (Colaprico et al., 2016) to download the gene expression data which including 371 HCC and 50 normal tissues in patients from TCGA data resource^1^ (up to October 19, 2019). The details have been listed in Supplementary Table S1.

For the raw data (.CEL files) detected by the Affymetrix platform, the RMA (Robust Multi-array Average) algorithm was used for background adjustment. If a gene was matched to multiple probes, the arithmetic mean expression value was used as the gene expression level. For the data sets detected by the Illumina platforms, we directly used the processed expression data.

### The Within−Sample Relative Expression Orderings

Within a sample, the REOs of two genes (*a* and *b*) is expressed as *Ea* > *Eb* (or *Ea* < *Eb*) if gene *a* has higher (or lower) expression level than gene *b*. The REOs pattern of a gene pair is regarded as stable if the REOs kept in at least 95% of the samples. A reversal gene pair is a gene pair with stable REOs in both cirrhosis tissues in patients without HCC (CwoHCC) samples and HCC samples, but the REOs patterns are reversed in the second group (*Ea* < *Eb* or *Ea* > *Eb* in CwoHCC samples but *Ea* > *Eb* or *Ea* < *Eb* in HCC samples). Here, the reversal gene pairs are selected as the candidate REOs signature for the identification of HCC. Then we obtained the common genes between training datasets and validation datasets and its corresponding gene expression profile. Subsequently, based on the gene expression profiles and reversal gene pairs, we generate a new profile by using 1, 0, and −1 to represent *Ea* > *Eb*, *Ea* < *Eb*, and other cases (*Ea* or *Eb* do not exist), respectively.

### Feature Selection Through mRMR and IFS Methods

Based on the new profiles, mRMR (minimum Redundancy Maximum Relevance) (Peng et al., 2005) was applied to ranking the gene pairs based on the conditions of maximum relevance with the disease type along with minimum redundancy with other gene pairs.

Here, Ω represents all 857 gene pairs, *gi* is a gene pair from the 857 gene pairs and *T* is the disease type. The mutual information (*I*) can be formulated as:

(1) $$I\,\left(g_{i},T\right)=\int p\,\left(g_{i},T\right)\,\ln\left(\frac{p\,\left(g_{i},T\right)}{p\,\left(g_{i}\right)\,p\,\left(T\right)}\right)\,dg_{i}\,dT.$$

The mRMR function:

(2) $$m\,R\,M\,R=\frac{1}{\left|\Omega \right|}\,\sum_{g_{i}\in \Omega }I\,\left(g_{i},T\right)-\frac{1}{{\left|\Omega \right|}^{2}}\,\sum_{g_{i}\,g_{j}\in \Omega }I\,\left(g_{i},g_{j}\right)$$

where *I(gi, T)* is mutual information between the *gi* gene pair and disease type *T*, *I(gi, gj)* is mutual information between *gi* and *gj*. Then we used incremental feature selection (IFS) (Tan et al., 2019; Yang et al., 2019) method to select the optimal gene pairs from 857 mRMR gene pairs as diagnostic signature. The details about IFS can be found in (Dao et al., 2019).

### Classification Through SVM

Support Vector Machine (SVM) is a powerful classification method which has been used extensively in the fields of biological data mining (Cao et al., 2014; Manavalan and Lee, 2017; Manavalan et al., 2017, 2018b,2019c,d; Tang et al., 2017; Bu et al., 2018; Zhang et al., 2018; Chao et al., 2019a, b; Wang et al., 2019). Here, the free package LibSVM (version 3.23) (Chang and Lin, 2011) was downloaded to implement SVM. Due to its good performance on non-linear problem, RBF (radial basis function) was utilized. The values of two parameters *C* and γ for SVM are determined by the use of grid search with fivefold cross-validation. In present work, the optimal values are *C* = 0.125 and γ = 0.5, respectively.

### Performance Metrics

The sensitivity, specificity and accuracy (Basith et al., 2019; Manavalan et al., 2018a, c, 2019a,b) was applied to evaluating the performance of prediction methods. Here, HCC samples were regarded as positive samples; CwoHCC samples were negative samples. Mathematical representation of the above mentioned measures are calculated as:

(3) $$\left\{ \begin{array}{c} \text{Sensitivity}=\frac{\text{TP}}{\text{TP}+\text{FN}}\\ \text{Specificity}=\frac{\text{TN}}{\text{TN}+\text{FP}}\\ \text{Accuracy}=\frac{\text{TP}+\text{TN}}{\text{TP}+\text{FP}+\text{TN}+\text{FN}} \end{array}\right.$$

where TP, FN, TN, and FP denotes the number of true positives, false negatives, true negatives, and false positives, respectively. Additionally, the ROC curve and AUC are commonly used to test the balance between true positive rate and false positive rate.

## Results

### Identification of the Diagnostic Signature

**FIGURE 1:**
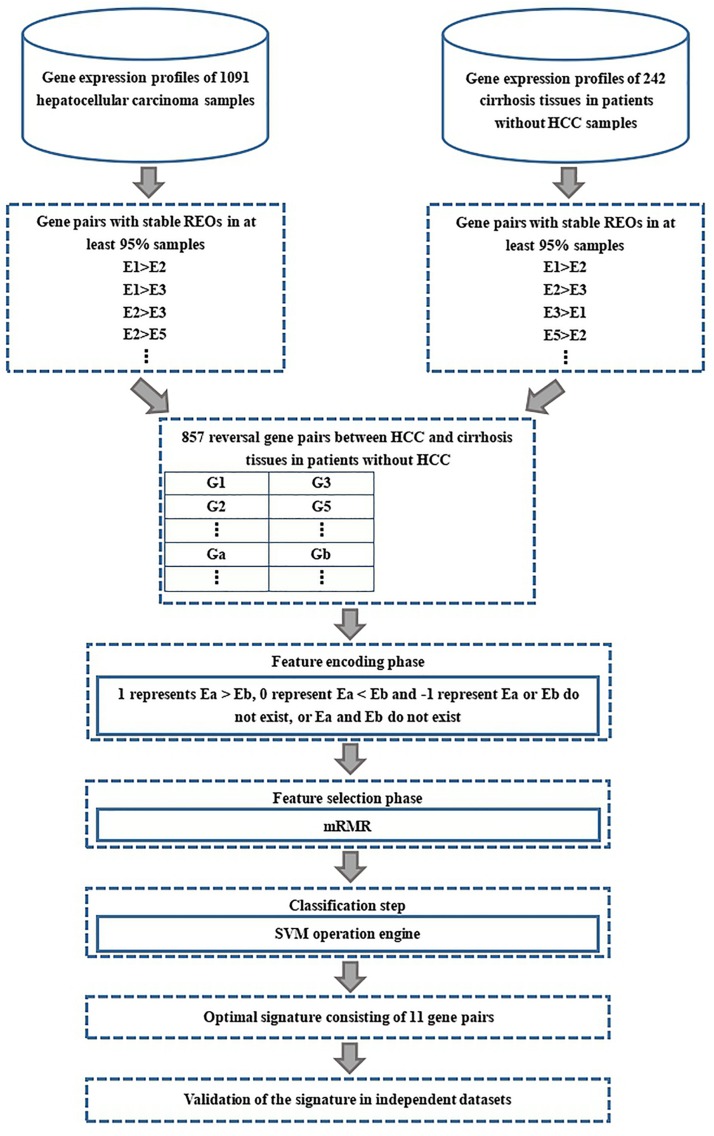
Flowchart presenting the process of developing and validating the HCC diagnostic signature.

**FIGURE 2:**
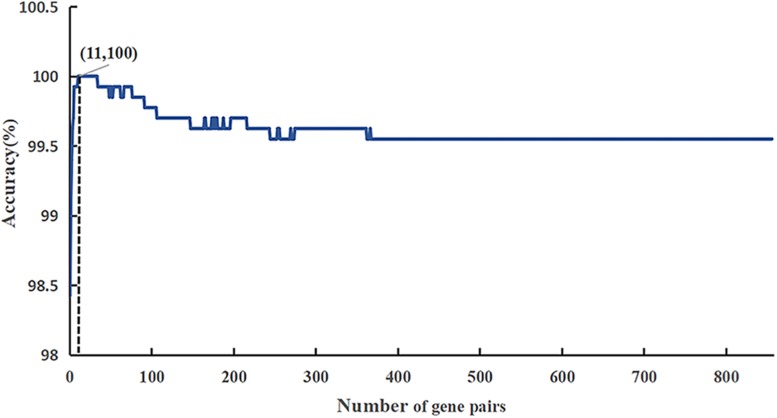
A plot showing the IFS procedure for identifying HCC. When the top 857 features optimized by mRMR were used to perform prediction, the overall success rate reaches an IFS peak of 100% in fivefold cross validation. The solid line represents the ROC curve. The dotted line represents the strategy of randomly guess.

The flow diagram for identifying and validating the diagnostic signature is shown in Figure 1. Firstly, total of 13,586,043 stable gene pairs which have an identical REOs in at least 95% of the 1091 HCC samples were recognized. Similarly, we also identified 14,475,509 stable gene pairs which have an identical REOs in at least 95% of the 242 CwoHCC samples. Then, we obtained 857 reversal gene pairs between the HCC samples and CwoHCC samples in the training data (see section “Materials and Methods”). Based on the new profiles (see section “Materials and Methods”), 11 gene pairs shown in Table 1 were picked out by using mRMR with SVM and regarded as the diagnostic signature. The 11-gene-pair could produce the accuracy of 100% on training data for HCC identification. Figure 2 showed the IFS process (blue curve).

**TABLE 1:** The 11−gene−pair signature for early diagnosis of HCC.

Signature	Gene *a*	Gene *b*
pair1	TRMT112	SF3B1
pair2	MFSD5	COLEC10
pair3	FDXR	APC2
pair4	LAMC1	CHST4
pair5	UBE4B	HGF
pair6	NCAPH2	APC2
pair7	HSPH1	MTHFD2
pair8	TMEM38B	AGO3
pair9	PLGRKT	COLEC10
pair10	HNF1A	APC2
pair11	ARPC2	SF3B1

*Gene *a* has a higher expression level than Gene *b* in HCC patients compared with CwoHCC patients.*

### Examination of the Diagnostic Signature on Independent Datasets

**FIGURE 3:**
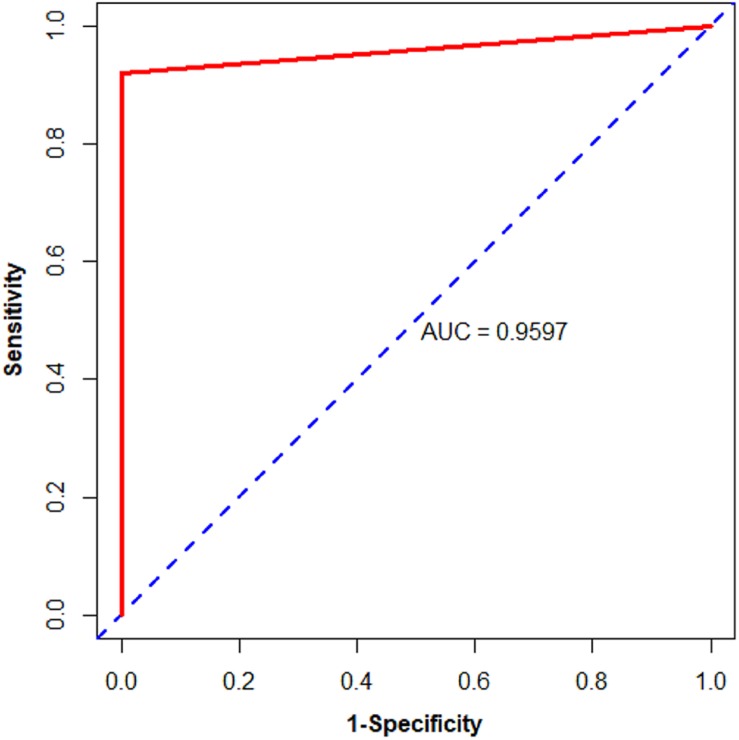
Area under the receiver operating characteristic curve (AUC) of the validation data from public databases of biopsy and surgically resected HCC and CwoHCC samples. The solid line represents the ROC curve. The dotted line represents the strategy of randomly guess.

Subsequently, we used biopsy and surgically resected samples to estimate the performance of the 11-gene-pair (see Table 2). For 73 biopsy samples in the testing datasets, it yielded accuracy of 100%, sensitivity of 100%, specificity of 100%. For 263 surgically resected samples in the testing datasets, its accuracy is 100%, sensitivity 100%, specificity 100%. In the data set GSE121248, all (100.0%) of the 70 HCC samples were correctly recognized as HCC. For surgically resected samples, 79.79% of the 475 HCC samples from 3 datasets (GSE109211, GSE112790, and GSE102079) were correctly classified. Moreover, the 11-gene-pair based model could correctly identify the 371 HCC and the 50 normal tissues in patients with HCC (NwHCC) samples measured by RNA-seq, in which no RNA-seq information was included (Table 2). These results demonstrated that the 11-gene-pair signature could distinguish HCC from non-cancerous liver tissues and the signature was robust to clinicopathological variations. For the 1190 HCC samples and 62 CwoHCC samples, the sensitivity, specificity, and AUC are 91.93%, 100%, and 0.9597 [95% CI (confidence intervals) is 0.9519–0.9674; see in Figure 3], respectively.

**TABLE 2:** The performance of the signature in the validation datasets.

Datasets	NSnHCC	NSpCwoHCC
Testing datasets (biopsy)	100%(29/29)	100% (44/44)
Testing datasets (surgery)	100%(245/245)	100% (18/18)
GSE109211	31.43%(44/140)	–
GSE112790	100%(183/183)	–
GSE102079	100%(152/152)	–
GSE121248	100%(70/70)	–
TCGA	100%(371/371)	–

*NSnHCC, number (sensitivity) of HCC samples; NSpCwoHCC, number (specificity) of CwoHCC samples.*

For biopsy samples, all of 80 cirrhosis tissues in patients with HCC (CwHCC) samples in GSE54236 and all of 97 NwHCC biopsy tissues from 2 datasets (GSE64041 and GSE121248) were correctly classified to HCC. The results proved again that, the 11-gene-pair still displayed good performance that most of HCC adjacent non-cancerous patients (CwHCC and NwHCC) can be correctly recognized, even for the inaccurate samples from biopsy specimens. For surgically resected samples, 93.7% of the 254 CwHCC samples and 100% of the 644 NwHCC samples can be accurately identified (see in Table 3). All above results demonstrated again that the obtained 11-gene-pair could be regarded as key biological signatures to diagnose HCC patients.

**TABLE 3:** Comparison of 11 gene pairs with existing methods on independent datasets.

Dataset	11-gene-pair			19-gene-pair		
		
	NSnHCC	NACwHCC	NANwHCC	NSnHCC	NACwHCC	NANwHCC
**Datasets from surgical resection**						
GSE6764	−	10/10(100.0%)	−	−	10/10(100.0%)	−
GSE17548	−	18/20(90.0%)	−	−	18/20(90.0%)	−
GSE17967	−	16/16(100.0%)	−	−	8/16(50.0%)	−
GSE63898	−	168/168(100.0%)	−	−	168/168(100.0%)	−
GSE25097	−	40/40(100.0%)	243/243(100.0%)	−	40/40(100.0%)	243/243(100.0%)
GSE62232	−	−	10/10(100.0%)	−	−	10/10(100.0%)
GSE36376	−	−	193/193(100.0%)	−	−	172/193(89.1%)
GSE39791	−	−	72/72(100.0%)	−	−	71/72(98.6%)
GSE41804	−	−	20/20(100.0%)	−	−	20/20(100.0%)
GSE112790	183/183(100.0%)	−	15/15(100.0%)	183/183(100.0%)	−	15/15(100.0%)
GSE102079	152/152(100.0%)	−	91/91(100.0%)	152/152(100.0%)	−	91/91(100.0%)
GSE109211	44/140(31.4%)	−	−	37/140(26.4%)	−	−
Total	379/475(79.8%)	238/254(93.7%)	644/644(100.0%)	372/475(79.3%)	244/254(96.1%)	622/644(96.6%)
**Datasets from biopsy**						
GSE121248	70/70(100.0%)	−	37/37(100.0%)	70/70(100.0%)	−	37/37(100.0%)
GSE64041	−	−	60/60(100.0%)	−	−	60/60(100.0%)
GSE54236	−	80/80(100.0%)	−	−	62/80(77.5%)	−
Total	70/70(100.0%)	80/80(100.0%)	97/97(100.0%)	70/70(100.0%)	62/80(77.5%)	97/97(100.0%)

*NACwHCC, number (accuracy) of cirrhosis tissues in patients with HCC samples to HCC; NANwHCC, number (accuracy) of normal tissues in patients with HCC samples to HCC.*

### Comparison With Existing Methods

To further demonstrate the performance of our proposed signatures, we compared our method with 19-gene-pair-based models and recorded results in Table 3. An earlier work done by Ao et al. (2018) found that 19-gene-pair can be regarded as diagnostic signature to discriminate HCC and adjacent non-cancerous tissues (cirrhosis or normal) from CwoHCC. Their model could produce 99.69% of accuracy which is lower than that of our 11-gene-pair based model.

For biopsy samples, our proposed model could correctly identify the 70 HCC samples in GSE121248 and the 97 NwHCC biopsy tissues from 2 datasets (GSE64041 and GSE121248) with the accuracy of 100%. Moreover, all 80 CwHCC samples in GSE54236 can be predicted as HCC. Compared with the accuracy (77.5%) of 19-gene-pair based model, the accuracy of 11-gene-pare model could increase to 100%.

For surgically resected samples, based on the predictor of 11-gene-pair, 79.8% of the 475 HCC samples from 3 datasets (GSE109211, GSE112790, and GSE102079) and 93.7% of the 254 CwHCC samples from 5 datasets (GSE6764, GSE17548, GSE25097, GSE17967, and GSE63898) can be corrected as HCC. Moreover, the model can accurately predict the 644 NwHCC biopsy tissues integrated from 7 datasets (GSE25097, GSE62232, GSE36376, GSE39791, GSE41804, GSE112790, and GSE102079). Also, the sensitivity of HCC samples increases to 79.8% (19-gene-pair: 79.3%) and the accuracy of NwHCC samples to HCC increases to 100% (19-gene-pair: 96.6%). It can be seen from Table 3 that in the identification of both HCC and adjacent non-cancerous tissues (CwHCC and NwHCC) from CwoHCC by surgically resected samples, the 11-gene-pair based model displayed better performance than the 19-gene-pair based model, demonstrating that the 11-gene-pair-based model is quite promising in generating reliable results for the early HCC diagnosis.

The above results showed that the proposed 11-gene-pair-based model is powerful on both training datasets and independent datasets. This achievement can be attribute to using within-sample REOs and SVM.

## Discussion

Clinical practice has demonstrated that diagnosing the tumors in early stages is key to improve the survival of patient. Although pathology is used as a gold standard for HCC diagnosis, the histological analysis of the HCC biopsy specimen is influenced by the sampling location and tissue amount. In present work, a set of diagnostic signature including 11-gene-pair consisting of 18 genes was identified, which can be used to discriminate HCC and adjacent non-cancerous tissues (CwHCC and NwHCC) from CwoHCC individuals for the early HCC diagnosis.

Ten genes in the signature set, including LAMC1, UBE4B, HSPH1, HNF1A, SF3B1, APC2, CHST4, HGF, MTHFD2, and AGO3, might have a vital role during the hepatocarcinogenesis and are key genes for cancer. For instance, LAMC1 mRNA can promote the development of HCC by competing with miR-124 and supporting the excretion of CD151 (Yang et al., 2017). UBE4B can be used as a potential prognostic marker for HCC treatment due to its carcinogenic effect in human primary HCC (Zhang et al., 2016). Additionally, HNF1A is closely associated with HCC because the number of HNF1A increase when non-cancerous liver develops into high differentiate HCC (Wang et al., 1998). SF3B1 is a highly conserved spliceosomal protein in evolution (Eilbracht and Schmidt-Zachmann, 2001) and its expression increases significantly in liver HCC tissues. Serum anti-SF3B1 autoantibody is a potential diagnostic marker for HCC patients (Hwang et al., 2018). Reportedly, HSPH1 (Yang et al., 2015), APC2 (Ghosh et al., 2016), CHST4 (Gao et al., 2015), HGF (Unic et al., 2018), MTHFD2 (Liu et al., 2016), and AGO3 (Kitagawa et al., 2013) are closely related to HCC.

Subsequently, the 18 genes (11-gene-pair) were used for functional enrichment analysis by using Metascape^2^ (Tripathi et al., 2015) on the KEGG (Kyoto Encyclopedia of Genes and Genomes) pathways and GO (Gene Ontology) terms. In order to determine the significant terms, *p*-value < 0.05 and the number of enriched genes ≥3 were used as the statistical standard. Finally, 18 genes were significantly enriched in the “ribonucleoprotein complex biogenesis,” “positive regulation of cellular component biogenesis,” “lymphocyte activation,” and “chemotaxis” terms based on GO analysis, as well as “Pathways in cancer” according to KEGG analysis. The above analysis showed that the genes of the 11-gene-pair might have vital roles in the development and progression of HCC.

In current study, we showed that 11 gen pairs can be applied to accurately diagnose the tumors found in the liver. Further, we shall try to establish a user-friendly web-server for the proposed “11-gene-pair” model. In the future, we will apply other feature selection techniques and algorithms to further improve the diagnosis of cancers.

## Data Availability Statement

The datasets used in this study can be freely download from the GEO (https://www.ncbi.nlm.nih.gov/geo/) and TCGA (https://portal.gdc.cancer.gov/repository) repository.

## Author Contributions

HL designed the study and revised the manuscript. Z-MZ carried out all the data collection and drafted the manuscript. Z-MZ, J-XT, FW, F-YD, and Z-YZ performed the data analysis. All authors approved the final manuscript.

## Conflict of Interest

The authors declare that the research was conducted in the absence of any commercial or financial relationships that could be construed as a potential conflict of interest.

## Footnotes

1 https://portal.gdc.cancer.gov/repository

2 http://metascape.org

## Abbreviations

CwHCC: cirrhosis tissues in patients with HCC
CwoHCC: cirrhosis tissues in patients without HCC
HCC: hepatocellular carcinoma
IFS: incremental feature selection
mRMR: maximum redundancy minimum relevance
REOs: relative expression orderings
SVM: support vector machine.
