# Supplementary material for: Early Diagnosis of Hepatocellular Carcinoma Using Machine Learning Method
Source: Front Bioeng Biotechnol. 2020 Mar 27;8:254. doi: 10.3389/fbioe.2020.00254 (PMC7122481; doi:10.3389/fbioe.2020.00254)
Supplement: Supplementary file 1 [file Table_1.DOCX]

Supplementary Material

# **Supplementary Tables**

**Supplementary Table S1. The publicly available datasets used in the study**

| **Dataset** | **Platform** | **HCC** | **CwoHCC** | **CwHCC** | **NwHCC** | **Sampling method** |
| --- | --- | --- | --- | --- | --- | --- |
| Datasets used for identification of the diagnostic signature | | | | | | |
| GSE14323 | GPL571 | 38 | 41 | - | - | Surgery |
| GSE14520 | GPL570 | 225 | - | - | - | Surgery |
| GSE62232 | GPL570 | 81 | - | - | - | Surgery |
| GSE36376 | GPL10558 | 240 | - | - | - | Surgery |
| GSE39791 | GPL10558 | 72 | - | - | - | Surgery |
| GSE41804 | GPL570 | 20 | - | - | - | Surgery |
| GSE6764 | GPL570 | 35 | - | - | - | Surgery |
| GSE17548 | GPL570 | 17 | - | - | - | Surgery |
| GSE25097 | GPL10687 | 268 | - | - | - | Surgery |
| GSE17967 | GPL571 | - | 47 | - | - | Surgery |
| GSE63898 | GPL13667 | 228 | - | - | - | Surgery |
| GSE15654 | GPL8432 | - | 216 | - | - | Biopsy |
| GSE64041 | GPL6244 | 60 | - | - | - | Biopsy |
| GSE54236 | GPL6480 | 81 | - | - | - | Biopsy |
| Datasets used for evaluating the performance of the diagnostic signature | | | | | | |
| GSE6764 | GPL570 | - | - | 10 | - | Surgery |
| GSE17548 | GPL570 | - | - | 20 | - | Surgery |
| GSE17967 | GPL571 | - | - | 16 | - | Surgery |
| GSE63898 | GPL13667 | - | - | 168 | - | Surgery |
| GSE25097 | GPL10687 | - | - | 40 | 243 | Surgery |
| GSE62232 | GPL570 | - | - | - | 10 | Surgery |
| GSE36376 | GPL10558 | - | - | - | 193 | Surgery |
| GSE39791 | GPL10558 | - | - | - | 72 | Surgery |
| GSE41804 | GPL570 | - | - | - | 20 | Surgery |
| GSE112790 | GPL570 | 183 | - | - | 15 | Surgery |
| GSE102079 | GPL570 | 152 | - | - | 91 | Surgery |
| GSE109211 | GPL13938 | 140 | - | - | - | Surgery |
| Total |  | 475 |  | 254 | 644 |  |
| GSE121248 | GPPL570 | 70 | - | - | 37 | Biopsy |
| GSE64041 | GPL6244 | - | - | - | 60 | Biopsy |
| GSE54236 | GPL6480 | - | - | 80 | - | Biopsy |
| Total |  | 70 |  | 80 | 97 |  |
| TCGA | HTSeq-FPKM | 371 | - | - | 42 | Surgery |

CwoHCC and CwHCC denote cirrhosis tissues in patients with HCC and cirrhosis tissues in patients without HCC respectively. NwHCC denotes normal tissues in patients with HCC.
